# Effects of particulate air pollution on blood pressure in a highly exposed population in Beijing, China: a repeated-measure study

**DOI:** 10.1186/1476-069X-10-108

**Published:** 2011-12-21

**Authors:** Andrea Baccarelli, Francesco Barretta, Chang Dou, Xiao Zhang, John P McCracken, Anaité Díaz, Pier Alberto Bertazzi, Joel Schwartz, Sheng Wang, Lifang Hou

**Affiliations:** 1 Department of Environmental Health, Harvard School of Public Health, Boston, Massachusetts, USA; 2 Department of Occupational and Environmental Health, University of Milan and Fondazione IRCCS Ca' Granda Policlinico, Milan, Italy; 3 Deptartment of Safety Engineering, China Institute of Industrial Health No.45 Zengguang Rd., Haidian District, Beijing 100048, China; 4 Department of Preventive Medicine, Feinberg School of Medicine, Northwestern University, Chicago, Illinois, USA; 5 Center for Health Studies, Universidad del Valle de Guatemala, Guatemala City, Guatemala; 6 Department of Occupational and Environmental Health, Peking University Health Science Center, Beijing, China

**Keywords:** Particulate Matter, Personal Monitoring, Blood Pressure, Traffic Pollution, China

## Abstract

**Background:**

Particulate Matter (PM) exposure is critical in Beijing due to high population density and rapid increase in vehicular traffic. PM effects on blood pressure (BP) have been investigated as a mechanism mediating cardiovascular risks, but results are still inconsistent. The purpose of our study is to determine the effects of ambient and personal PM exposure on BP.

**Methods:**

Before the 2008 Olympic Games (June 15-July 27), we examined 60 truck drivers and 60 office workers on two days, 1-2 weeks apart (n = 240). We obtained standardized measures of post-work BP. Exposure assessment included personal PM_2.5 _and Elemental Carbon (EC, a tracer of traffic particles) measured using portable monitors during work hours; and ambient PM_10 _averaged over 1-8 days pre-examination. We examined associations of exposures (exposure group, personal PM_2.5_/EC, ambient PM_10_) with BP controlling for multiple covariates.

**Results:**

Mean personal PM_2.5 _was 94.6 μg/m^3 ^(SD = 64.9) in office workers and 126.8 (SD = 68.8) in truck drivers (p-value < 0.001). In all participants combined, a 10 μg/m^3 ^increase in 8-day ambient PM_10 _was associated with BP increments of 0.98 (95%CI 0.34; 1.61; p-value = 0.003), 0.71 (95%CI 0.18; 1.24; p-value = 0.01), and 0.81 (95%CI 0.31; 1.30; p-value = 0.002) mmHg for systolic, diastolic, and mean BP, respectively. BP was not significantly different between the two groups (p-value > 0.14). Personal PM_2.5 _and EC during work hours were not associated with increased BP.

**Conclusions:**

Our results indicate delayed effects of ambient PM_10 _on BP. Lack of associations with exposure groups and personal PM_2.5_/EC indicates that PM effects are related to background levels of pollution in Beijing, and not specifically to work-related exposure.

## Background

Epidemiologic studies have consistently associated short-term increases in exposure to air particles with higher rates of hospitalization and mortality for cardiovascular disease in the hours and days following exposure peaks [1]. Airborne particulate matter ≤2.5 μm (PM_2.5_) or ≤10 μm (PM_10_) in aerodynamic diameter can be inhaled and deposited in the upper and lower airways [2]. Several pathways have been proposed to link PM inhalation with these acute cardiovascular effects, including inflammatory, endothelial, and autonomic responses [1]. However, the patho-physiological changes linking air pollution inhalation to cardiovascular events have not been fully elucidated. Elevated BP is an established risk factor for coronary heart disease and stroke, and may be implicated in the association of short-term PM exposure with cardiovascular disease. An increase as small as 1 mmHg in usual systolic BP is estimated to increase by 2-4% the risk of death due to cardiovascular disease [3,4]. Studies have examined air particle exposures in relation to BP elevation with results showing several positive [5-14], but also some negative [15-18] and null associations [19-21]. Several of the previous investigations did not have BP as the primary outcome and as such were not designed with the explicit intention to evaluate the association between PM and BP [1].

Beijing has been ranked as one of the 15 cities with the highest levels of air pollution worldwide [22]. Traffic-derived PM is critical in Beijing due to very high population density and rapid increase in vehicular traffic [23]. Transported particles from industrial sources and windblown dust are also major sources of pollution [23]. Examining the effects of high levels of PM such as those found in Beijing may help to characterize changes in BP that might not be consistently demonstrated in populations with lower exposures.

In the present study, we investigated 60 truck drivers and 60 indoor workers in Beijing to evaluate whether either typical or short-term exposure to air particles is associated with effects on BP. To enhance power to identify effects on BP, we studied each subject on two different examination days, 1-2 weeks apart, and assessed exposure using personal measures of PM_2.5 _and Elemental Carbon (EC, a surrogate for traffic particles) on the day of the exam and ambient levels of PM_10 _up to eight days before the exam.

## Methods

### Study population and design

The Beijing Truck Driver Air Pollution Study (BTDAS) was conducted between June 15 to July 27, 2008, shortly before the Beijing Olympic Games. The BTDAS included 60 truck drivers and 60 indoor office workers. Because PM levels are highly variable on a day-to-day basis, we examined all subjects on two workdays separated by 1-2 weeks. Both truck drivers and office workers worked and lived in the Beijing metropolitan area and had been on their current jobs for ≥ two years. The two groups were matched by sex, smoking status and education, and partially matched (5-year intervals) by age. In-person questionnaire-based interviews were conducted to collect information on demographics, lifestyle, and other exposures. Information on time-varying factors, including tea, alcohol, and smoking, was obtained for past usual exposure, as well as on each examination day. Individual written informed consent and Institutional Review Board approval was obtained prior to the study.

### Personal exposure measurements

**Figure 1:**
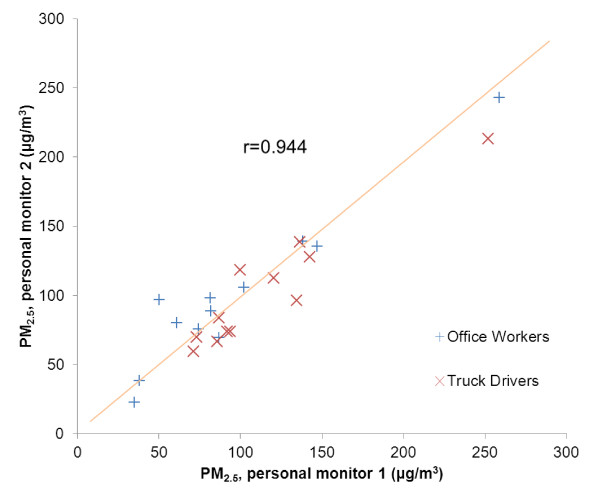
**Measures of PM_2.5 _from two independent personal monitors**. Measures of PM_2.5 _from two independent personal monitors worn at the same time by a subset of 12 study subjects to test the accuracy of the measurements. The scatter plot shows the high correlation (r = 0.944) between monitor 1 and monitor 2.

We measured personal PM_2.5 _on both examination days using gravimetric samplers worn by the study subjects during the eight hours of work. The sampler was carried in a belt pack with the inlet clipped near the breathing zone. Each sampler setup included an Apex pump (Casella Inc., Bedford, UK), a Triplex Sharp-Cut Cyclone (BGI Inc., Waltham, Massachusetts), and a 37-mm Teflon filter placed on top of a drain disc and inside a metal filter holder. The filters were kept under atmosphere-controlled conditions before and after sampling and were weighed with a microbalance (Mettler-Toledo Inc., Columbus, Ohio, USA). A time-weighted average of PM_2.5 _concentration was recorded by dividing the change in filter weight before and after sampling by the volume of air sampled. We found high reproducibility of PM_2.5 _measures (r = 0.944) in replicate measures on a subset of 24 subjects who wore two monitors at the same time (Figure 1). The blackness of the same filters used to measure PM_2.5 _was assessed using an EEL Model M43D Smokestain Reflectometer, applying the standard black-smoke index calculations of the absorption coefficients based on reflectance [24]. We assumed a factor of 1.0 for converting the absorption coefficient to EC mass [25,26], which was then divided by the sampled air volume to calculate average EC exposure concentration [24]. EC is a combustion by-product contained in PM that has been used as a surrogate measure for PM from gasoline- and especially diesel-powered motor vehicles [25].

### Ambient PM_10 _data

Ambient PM_10 _data during the study period were obtained from the Beijing Municipal Environmental Bureau (http://www.bjepb.gov.cn/air2008/Air.aspx). We used daily averages of PM_10 _computed from data obtained from 27 monitoring stations to estimate the average PM_10 _level in Beijing. The monitoring stations are distributed across the area to represent Beijing city. We used ambient PM_10 _data to test the hypothesis that the association between particles and blood pressure is with a longer-term average exposure than with the personal monitors. We used multiple averaging time windows, which included 1-day mean (24 hour average of the day before the examination), as well as 2-day, 5-day, and 8-day means (i.e., average of the 2-8 days before the examination). We obtained daily outdoor temperature data for Beijing city from the National Oceanic and Atmospheric Administration online database [27].

### Seated BP and heart rate measurements

Seated BP and heart rate were measured by a trained research assistant at the end of each work day (i.e., between 4-6 pm) after a full five minutes of rest. Heart rate was taken in the sitting position by measuring it over a 30-second period by pulse palpation at the radial artery. Two heart rate measurements were taken and their average was recorded. A standardized protocol for BP measurements was used according to the recommendations issued by the American Heart Association [28]. BP was measured using a mercury sphygmomanometer on the right arm using appropriate cuff sizes. All readings were made to the nearest even digit by rounding up if necessary. Three readings were taken and BP was calculated from the average of the second and third readings. After each reading, the research assistant waited at least one minute before proceeding to the next reading. Mean arterial pressure was approximated from systolic and diastolic BP by adding 1/3 of the difference between systolic and diastolic BP to the value of diastolic BP. Pulse pressure was defined as the difference between systolic and diastolic BP.

### Statistical analysis

Standard descriptive statistics were used to describe the characteristics of truck drivers and office workers. For variables considered constant within-subjects between the two examination days, such as age, sex, and usual smoking habits, differences in participant characteristics between the two groups were tested using Student's t-tests and Fisher's exact tests. For variables that varied between the two examination days, such as tea consumption or number of cigarettes smoked on that day, we evaluated differences between the two groups using mixed-effect regression models (PROC MIXED in SAS 9.2, SAS Institute Inc., Cary, NC). Similarly, we used mixed-effect models to regress BP or heart rate variables on group (0, office workers; 1, truck drivers) to test for differences between groups and estimate group-specific means and standard deviations (SDs). For BP or heart rate variables, we fitted unadjusted models as well as models adjusted for variables either not matched or not completely matched by design between the two groups, i.e. age (continuous), BMI (continuous), cigarettes smoked during study time (continuous), pack-years of smoking (continuous), tea consumption during study time (yes/no), usual alcohol drinking (yes/no), work hours/week (continuous) and day of the week (one indicator variable per day). The mixed-effect regression models were:

$$y_{ij}=\beta_{0}+\beta_{1}\left(\text{Group}\right)+\beta_{2}X_{2j}+...+\beta_{n}X_{nj}+\xi_{ij}+e_{ij}$$

where β_0 _is the overall intercept; β_1 _is the regression coefficient for the group; β_2_... β_n _are the regression coefficients for the covariates included in multivariate models; ξ*_ij _*is the random effect for the subject; *j *represents the subject; *i *identifies the workday and *e_ij _*is the residual error term.

We evaluated the associations of personal PM_2.5_, personal EC, and ambient PM_10 _variables (1-day, 2-day, 5-day, or 8-day mean) with BP variables or heart rate using mixed-effect models adjusted for age (continuous), sex (male, female), BMI (continuous), day of the week (one indicator variable per day), smoking (never, former, current), cigarettes smoked during study time (continuous), pack-years of smoking (continuous), work hours/week (continuous), tea consumption during study time (yes/no), usual alcohol drinking (yes/no) and outdoor temperature (continuous). To optimize power, we conducted primary analyses on the association of exposure measures and BP or heart rate by fitting these models in all participants combined. Secondarily, we evaluated associations in office workers or truck drivers separately. For outdoor temperature, we used averaging times (one to eight days) to match the averaging times used for the air particle variables. The mixed-effect model was:

$$y_{ij}=\beta_{0}+\beta_{1}{\left(Exp\right)}_{i}+\beta_{2}({\text{Temp)}}_{i}+\beta_{3}{X_{3}}_{i}+...+\beta_{n}X_{ni}+\xi_{ij}+e_{ij}$$

where β_0 _is the overall intercept; β_1 _is the regression coefficient for exposure variable (EC, PM_2.5, _or PM_10_); β_2 _is the regression coefficient of the mean temperature of the days of interest; β_3_... β_n _are the regression coefficients for the covariates included in multivariate models; ξ*_ij _*is the random effect for the subject; *j *represents the subject; *i *represents the examination day, and *e_ij _*is the residual error term. All tests were two-sided and an alpha level of less than 0.05 was considered significant.

## Results

### Characteristics of the study participants

The characteristics of the 60 office workers and 60 truck drivers are shown in Table 1. Truck drivers were moderately, but significantly older than office workers. Truck drivers had higher BMI, reported a higher number of pack-years of smoking, smoked more cigarettes during the study time, and included a higher proportion of usual alcohol drinkers. A larger proportion of truck drivers reported tea consumption during the study period.

**Table 1:** Characteristics of the Study Participants

		Office Workers (n = 60)	Truck Drivers (n = 60)	p-value^a^
**Sex, n (%)**				

	Male	40 (66.67)	40 (66.67)	

	Female	20 (33.33)	20 (33.33)	1.00

**Age [Years], mean ± SD**		30.27 ± 7.96	33.53 ± 5.65	0.004

**Smoking, n (%)**				

	Never smoker	35 (58.33)	34 (56.67)	

	Ex-smoker	2 (3.33)	2 (3.33)	

	Actual smoker	23 (38.33)	24 (40)	1.00

**Pack-years of smoking [kg/m^2^], mean ± SD^b^**		2.87 ± 3.59	11.7 ± 11.2	<0.001

**Cigarettes smoked during the study time^c^ [cigarettes/day], mean ± SD**		2.85 ± 5.21	6.39 ± 9.41	<0.001**^c^**

**BMI [kg/m^2^], mean ± SD**		22.76 ± 3.38	24.27 ± 3.21	0.01

**Tea consumption during the time of the study^c^, n (%)**				

	No	109 (90.83)	86 (71.67)	

	Yes	11 (9.17)	34 (28.33)	0.003**^c^**

**Day of the week^c^, n (%)**				

	Monday	16 (13.33)	19 (15.83)	

	Tuesday	18 (15)	13 (10.83)	

	Wednesday	14 (11.67)	15 (12.5)	

	Thursday	15 (12.5)	20 (16.67)	

	Friday	17 (14.17)	19 (15.83)	

	Saturday	18 (15)	16 (13.33)	

	Sunday	22 (18.33)	18 (15)	0.88**^c^**

**Usual alcohol drinking, n (%)**				

	Yes	14 (23.33)	31 (51.67)	

	No	46 (76.67)	29 (48.33)	0.002

^a^P-values were calculated using Student's t-test and Fisher's exact test for continuous and categorical variables, respectively, except for the variables indicated at the footnote ^c ^below.

^b^Only current or former smokers.

**^c^**Cumulative of the two study days. Based on 240 total observations (120 study days for office workers and 120 study days for truck drivers). P-values were obtained from mixed-effect regression models.

### Personal exposure and ambient levels of air particles

Table 2 shows the levels and distribution of personal time-weighted average exposure to PM_2.5 _and EC estimated during eight work hours, as well as the mean levels of ambient PM_10 _on the days before the examination days. Average personal PM_2.5 _was 126.8 μg/m^3 ^in truck drivers and 94.6 μg/m^3 ^for office workers (p-value < 0.001). Average personal EC was 17.2 μg/m^3 ^in truck drivers and 13.0 μg/m^3 ^for office workers (p-value < 0.001). As expected, the levels of ambient PM_10 _in the city of Beijing on the days before the examinations (1-8 day means) did not differ between truck drivers and office workers (Table 2).

**Table 2:** Levels of personal exposure to PM_2.5_ and Elemental Carbon (EC) during work hours, and of ambient PM_10_ and outdoor temperature on the days before examination

	Time window	Office Workers								Truck Drivers								
		**N**	**Mean**	**SD**	**10pct**	**25pct**	**Median**	**75pct**	**90pct**	**N**	**Mean**	**SD**	**10pct**	**25pct**	**Median**	**75pct**	**90pct**	**p-value**

**Personal PM_2.5_^a^ (μg/m^3^) on the examination days, from personal monitors**																		

	8 hours	120	94.6	64.9	22.4	48.5	86.2	126.6	183.4	119	126.8	68.8	46.3	73.9	116.8	160.5	213.9	<0.001

																		

**Personal EC^a^ (μg/m^3^) on the examination days, from personal monitors**																		

	8 hours	118	13.0	4.0	7.1	10.0	13.2	15.8	18.4	120	17.2	6.6	9.2	12.9	16.7	20.9	26.1	<0.001

																		

**Ambient PM_10_ (μg/m^3^) from ambient monitors on the days prior to the study days**																		

	1-day mean	120	121.5	47.8	72.0	82.0	118.0	146.0	186.0	120	119.5	51.2	64.0	82.0	118.0	142.0	188.0	0.76

	2-day mean	120	121.6	38.0	74.5	93.0	125.0	146.0	173.0	120	119.3	40.3	66.0	91.0	120.0	144.0	157.0	0.64

	5-day mean	120	119.5	26.9	80.7	105.6	119.6	138.0	148.8	120	118.2	25.6	81.0	96.8	119.6	136.8	144.0	0.69

	8-day mean	120	119.5	23.0	84.9	101.8	119.9	141.5	146.5	120	120.2	21.5	95.6	102.8	120.4	139.0	146.3	0.81

																		

**Outdoor temperature (°C) on the days prior to the study days**																		

	1-day mean	120	25.1	2.7	22.0	23.0	26.0	28.0	29.0	120	25.3	2.6	22.0	23.0	26.0	28.0	29.0	0.75

	2-day mean	120	25.2	2.3	22.0	23.0	25.5	27.5	28.0	120	25.0	2.7	22.0	23.0	25.3	27.0	28.0	0.56

	5-day mean	120	25.1	1.8	22.6	23.6	25.6	26.4	27.2	120	24.9	1.7	22.6	23.2	25.4	26.4	27.0	0.30

	8-day mean	120	25.0	1.4	23.1	24.0	24.6	26.4	27.0	120	24.9	1.4	23.1	23.6	24.5	26.3	26.9	0.34

^a^Measured during the work hours of examination days using light-weight personal monitors.

### Blood pressure and heart rate in truck drivers and office workers

In unadjusted analyses, truck drivers showed higher diastolic BP than office workers (p-value = 0.03), but no significant differences in systolic BP, mean arterial BP, pulse pressure, and heart rate (Table 3). Analyses adjusted by age, BMI, pack-years of smoking, number of cigarettes smoked and tea consumption during the time of the study, usual alcohol drinking, day of the week, and work hours/week did not show any statistically significant difference in systolic, diastolic, mean, or heart rate (Table 3). In the covariate-adjusted model, average pulse pressure in truck drivers was marginally higher than in office workers (p-value = 0.07).

**Table 3:** Blood pressure and heart rate in office workers and truck drivers

	Office Workers		Truck Drivers		
	**N**	**Mean ± SD**	**N**	**Mean ± SD**	**p-value**

**Unadjusted**					

Systolic blood pressure (mmHg)	120	115.3 ± 11.7	120	116.3 ± 13.3	0.56

Diastolic blood pressure (mmHg)	120	77.6 ± 8.3	120	80.2 ± 9.7	0.03

Mean Arterial pressure (mmHg)	120	90.2 ± 8.6	120	92.3 ± 10.5	0.10

Pulse pressure (mmHg)	120	37.7 ± 9.0	120	36.1 ± 7.4	0.13

Heart Rate (beats/min)	120	78.3 ± 10.4	120	79.3 ± 10.9	0.49

					

**Adjusted for age, BMI, pack-years, number of cigarettes and tea consumption during the time of the study, usual alcohol drinking, work hours/week, and day of the week^a^**					

Systolic blood pressure (mmHg)	120	118.9 ± 1.7	120	115.4 ± 1.5	0.14

Diastolic blood pressure (mmHg)	120	79.9 ± 1.2	120	79.2 ± 1.1	0.70

Mean Arterial pressure (mmHg)	120	92.8 ± 1.2	120	91.2 ± 1.1	0.36

Pulse pressure (mmHg)	120	39.3 ± 1.2	120	36.3 ± 1.1	0.07

Heart Rate (beats/min)	120	78.9 ± 1.5	120	79.6 ± 1.3	0.72

^a^Office workers and truck drivers were matched by sex and smoking (never, former, current). Adjusted means were computed by holding covariates fixed at their average values.

### Associations of personal PM_2.5_, personal EC, and ambient PM_10 _with blood pressure and heart rate

In analyses conducted on all participants combined, personal PM_2.5 _and EC measured during work hours did not show any significant association with BP measures or heart rate (Table 4). Also, the levels of ambient PM_10 _on the day before the examinations were not significantly associated with BP measures or heart rate. In all participants combined, BP increased in association with the levels of ambient PM_10 _averaged over five or eight days before the examinations. A 10 μg/m^3 ^increase in the 5-day mean of ambient PM_10 _was associated with an average increase of 0.63 mmHg in systolic BP (95%CI 0.09; 1.16; p-value = 0.02), 0.50 mmHg in diastolic BP (95%CI 0.06; 0.95; p-value = 0.03), and 0.55 mmHg in mean arterial pressure (95%CI 0.13; 0.96; p-value = 0.01). A 10 μg/m^3 ^increase in the 8-day mean of ambient PM_10 _was associated with an average increase of 0.98 mmHg in systolic BP (95%CI 0.34; 1.61; p-value = 0.003), 0.71 mmHg in diastolic BP (95%CI 0.18; 1.24; p-value = 0.01), and 0.81 mmHg in mean arterial pressure (95%CI 0.31; 1.30; p-value = 0.002). In all subjects combined, personal PM_2.5_, personal EC, and ambient PM_10 _were not associated with heart rate (Table 4).

**Table 4:** Effects of a 10 μg increase in air particles on blood pressure and heart rate, by group and on all subjects combined^a^

	All Subjects (obs = 240^b^)			Office Workers (obs = 120^c^)			Truck Drivers (obs = 120^d^)		
	**β**	**(95%CI)**	**p-value**	**β**	**(95%CI)**	**p-value**	**β**	**(95%CI)**	**p-value**

**Systolic blood pressure (mmHg)**									

Personal PM_2.5_ (work hours)	-0.01	(-0.18;0.17)	0.94	-0.06	(-0.29;0.18)	0.64	0.15	(-0.16;0.46)	0.33

Personal EC (work hours)	-0.29	(-2.32;1.73)	0.77	-2.54	(-6.39;1.31)	0.19	1.23	(-1.53;3.99)	0.38

Ambient PM_10 _ (1-day mean)	0.20	(-0.05;0.45)	0.11	0.10	(-0.26;0.46)	0.57	0.24	(-0.13;0.60)	0.20

Ambient PM_10 _ (2-day mean)	0.26	(-0.08;0.59)	0.14	-0.05	(-0.53;0.44)	0.85	0.47	(-0.04;0.97)	0.07

Ambient PM_10 _ (5-day mean)	0.63	(0.09;1.16)	0.02	0.08	(-0.80;0.95)	0.86	0.97	(0.15;1.78)	0.02

Ambient PM_10 _ (8-day mean)	0.98	(0.34;1.61)	0.003	0.53	(-0.44;1.50)	0.28	1.31	(0.32;2.31)	0.01

**Diastolic blood pressure (mmHg)**									

Personal PM_2.5_ (work hours)	0.04	(-0.11;0.19)	0.57	0.00	(-0.21;0.22)	0.97	0.09	(-0.14;0.33)	0.42

Personal EC (work hours)	-1.26	(-2.94;0.43)	0.14	-4.52	(-7.87;-1.16)	0.01	0.23	(-1.84;2.3)	0.83

Ambient PM_10 _ (1-day mean)	0.18	(-0.03;0.39)	0.09	0.11	(-0.21;0.43)	0.49	0.24	(-0.03;0.51)	0.08

Ambient PM_10 _ (2-day mean)	0.17	(-0.11;0.46)	0.23	0.06	(-0.38;0.50)	0.78	0.15	(-0.22;0.53)	0.42

Ambient PM_10 _ (5-day mean)	0.50	(0.06;0.95)	0.03	0.31	(-0.43;1.06)	0.40	0.34	(-0.29;0.97)	0.28

Ambient PM_10 _ (8-day mean)	0.71	(0.18;1.24)	0.01	0.83	(0.02;1.64)	0.04	0.07	(-0.72;0.87)	0.86

**Mean arterial pressure (mmHg)**									

Personal PM_2.5_ (work hours)	0.03	(-0.11;0.17)	0.66	-0.01	(-0.19;0.18)	0.95	0.12	(-0.11;0.35)	0.30

Personal EC (work hours)	-0.94	(-2.52;0.63)	0.24	-3.74	(-6.70;-0.78)	0.01	0.51	(-1.55;2.57)	0.62

Ambient PM_10 _ (1-day mean)	0.20	(0.01;0.39)	0.04	0.12	(-0.16;0.41)	0.39	0.25	(-0.02;0.51)	0.07

Ambient PM_10 _ (2-day mean)	0.20	(-0.07;0.46)	0.14	0.05	(-0.34;0.44)	0.81	0.25	(-0.13;0.62)	0.19

Ambient PM_10 _ (5-day mean)	0.55	(0.13;0.96)	0.01	0.27	(-0.42;0.95)	0.44	0.56	(-0.05;1.17)	0.07

Ambient PM_10 _ (8-day mean)	0.81	(0.31;1.30)	0.002	0.74	(0.00;1.48)	0.05	0.48	(-0.29;1.26)	0.22

**Pulse pressure (mmHg)**									

Personal PM_2.5_ (work hours)	-0.06	(-0.22;0.10)	0.49	-0.06	(-0.31;0.20)	0.65	0.06	(-0.20;0.31)	0.65

Personal EC (work hours)	0.75	(-1.12;2.61)	0.42	2.69	(-1.42;6.8)	0.19	0.98	(-1.18;3.14)	0.37

Ambient PM_10 _ (1-day mean)	0.01	(-0.22;0.23)	0.96	-0.03	(-0.40;0.33)	0.86	0.02	(-0.28;0.32)	0.90

Ambient PM_10 _ (2-day mean)	0.10	(-0.21;0.41)	0.51	-0.10	(-0.60;0.40)	0.68	0.33	(-0.08;0.75)	0.11

Ambient PM_10 _ (5-day mean)	0.12	(-0.38;0.61)	0.64	-0.26	(-1.13;0.60)	0.54	0.56	(-0.13;1.26)	0.11

Ambient PM_10 _ (8-day mean)	0.22	(-0.37;0.81)	0.46	-0.38	(-1.35;0.59)	0.44	1.08	(0.27;1.89)	0.01

**Heart rate (bpm)**									

Personal PM_2.5_ (work hours)	0.15	(-0.08;0.39)	0.20	0.00	(-0.31;0.30)	0.97	0.30	(-0.14;0.74)	0.18

Personal EC (work hours)	1.03	(-1.62;3.68)	0.44	-2.08	(-6.94;2.79)	0.40	2.19	(-1.52;5.89)	0.24

Ambient PM_10 _ (1-day mean)	0.14	(-0.19;0.48)	0.40	-0.03	(-0.48;0.41)	0.88	0.29	(-0.23;0.81)	0.27

Ambient PM_10 _ (2-day mean)	0.23	(-0.23;0.68)	0.33	-0.14	(-0.73;0.46)	0.64	0.61	(-0.13;1.35)	0.10

Ambient PM_10 _ (5-day mean)	0.57	(-0.14;1.29)	0.12	-0.13	(-1.14;0.89)	0.81	1.20	(-0.02;2.42)	0.05

Ambient PM_10 _ (8-day mean)	0.67	(-0.20;1.53)	0.13	-0.44	(-1.59;0.71)	0.45	1.69	(0.27;3.12)	0.02

^a^Adjusted for age, sex, BMI, smoking status, pack-years, number of cigarettes smoked and tea drinking during the study time, usual alcohol drinking, work hours/week, day of the week, and appropriate outdoor temperature (i.e., temperature averaged over the same time window as the air particle exposure variable).

^b^For EC, results are estimated on 238 observations because of two missing values; for PM_2.5_, results are from 239 observations because of one missing value.

^c^For EC exposure, results are estimated on 119 observations because of two missing values.

^d^For PM_2.5_ and EC exposures, results are estimated on 119 observations because of a missing value.

Separate analyses in office workers and truck drivers showed that the associations of the 5- and 8-day means of ambient PM_10 _with BP were found in each of the two groups (Table 4). Associations of ambient PM_10 _with systolic BP appeared moderately stronger in truck drivers (5-day and 8-day), whereas associations of ambient PM_10 _with diastolic BP appeared stronger in office workers, particularly for the 8-day mean (Table 4). In addition, in office workers we observed an unexpected negative association of personal EC levels with diastolic BP (p-value = 0.01) and mean arterial pressure (p-value = 0.01). In truck drivers, we found that the 8-day average ambient PM_10 _levels were associated with significant increases in pulse pressure (p-value = 0.01) and heart rate (p-value = 0.02). In truck drivers, the 5-day average ambient PM_10 _levels were also marginally associated with increased heart rate (p-value = 0.05). To evaluate the potential masking of air pollution effects by smoking, we conducted additional analyses stratified by current smoking. In the additional files, we report the results stratified by current smoking for the associations of personal PM_2.5_, personal EC, and ambient PM_10 _with BP and heart rate for the entire study group (**Table S1**, Additional file 1), as well as for office workers (**Table S2**, Additional file 1) or truck drivers (**Table S3**, Additional file 1). Overall, these analyses do not suggest that the effects of the exposures were different among current or non-current smokers.

## Discussion

In this study of truck drivers and office workers in Beijing, China, we showed increases in systolic, diastolic, and mean arterial BP associated with the levels of ambient PM_10 _averaged over five and eight days before the BP examination days. We found no significant positive associations of BP with personal measures of PM_2.5 _and EC taken during work hours on the day of the examination, nor with ambient PM_10 _averaged over 1-2 days before the examination days. Taken together, these results suggest that comparatively higher levels of PM exposure exert effects on BP that appear with a delay or possibly require 5-8 days to build up and become detectable. BP was higher among truck drivers than office workers, but there was no statistically significant difference after adjustment for potential confounders. Therefore, our results do not provide support for effects of work-related exposure to air particles on BP.

Previous studies that showed positive associations between PM exposure and BP estimated that a 10 μg/m^3 ^increase in PM_2.5 _is expected to raise BP by approximately 1-5 mmHg, as summarized by Brook and Rajagopalan [29]. In the present study, we found that a 10 μg/m^3 ^increase in average ambient PM_10 _in the eight days before the examinations was associated with increases in BP equal to 0.71-0.98 mmHg. These estimates need to be interpreted in the context of the exposure measures and PM levels found in Beijing. To estimate the effects of PM exposure in the days before the examinations, we used ambient data from the monitor network of the city of Beijing, which measures ambient PM_10_. PM_10 _contains both coarse particles, which are mostly filtered out in the upper airways, and fine and ultrafine particles which are considered to be primarily responsible for the cardiovascular effects of PM [1]. PM_2.5_, which is more widely measured in the North America and Europe, is considered a better measure of smaller particles and might more effectively help to capture PM effects [2]. A study that measured different PM fractions in Beijing in the summer of 2006 showed that PM_2.5 _represented approximately 60% of ambient PM_10 _[30]. Therefore, the use of ambient PM_10 _in our analysis might have contributed to reduce our effect estimates. If the effects that we observed were entirely due to the PM_2.5 _component, the estimated effect per 10 μg/m^3 ^increase in PM_2.5 _would be about 1.5 mmHg, which is well within the range of the summary above.

Moreover, the effect estimates, which we reported as changes in BP for each 10 μg/m^3 ^increase in PM_2.5 _or PM_10_, need to be considered against the absolute levels of PM exposure. For instance, the average levels of ambient PM_10 _in Beijing were approximately 120 μg/m^3 ^during our study. As a reference, the average urban-population weighted PM_10 _in the United States was 19 μg/m^3 ^in the year 2008 [22]. Therefore, due to the high concentrations and wide ranges of PM found in Beijing, even small BP changes for each 10 μg/m^3 ^increase in PM_10 _may correspond to comparatively high overall effects. However, it should also be noted that the dose-response slope between particles and cardiovascular mortality has been shown to be nonlinear, with lower slopes at higher particle concentrations [31]. Therefore, PM effects might be substantial at low to middle range doses and taper off at higher concentrations.

It is well established that increases in BP of similar magnitude to those that could be attributed to PM exposure in our study in Beijing substantially increase long-term risks of coronary and cerebrovascular events [4,32]. However, risks of these events are thought to be related to long-term elevations in BP [4,32]. Whether the shorter term effects on BP we observed might contribute to long-term cardiovascular risk or trigger acute cardiovascular events remains to be determined.

In our study, we found increases in BP only in association with the means of ambient PM_10 _over five or eight days before the examinations. However, we did not find any significant association of BP with the personal measures of PM_2.5_, which were taken during the 8-hour work shift immediately preceding the BP measures. Our results indicate delayed or cumulative effects of PM on BP. Consistent with our findings, most previous studies have shown that BP increases only days (lags two to five) after an elevation in ambient PM or even following a longer duration of higher exposure levels (up to 30 days) [29]. For instance, Ibald-Mulli et al. [8] showed a significant increase in systolic BP in a study of 2607 adults in Augsburg, Germany associated with the mean of total suspended particles in the previous five days. Zanobetti et al. [9] found significant increases in systolic and diastolic BP in cardiac rehabilitation patients related to the average PM_2.5 _in the previous five days. Effects on BP have been associated with 7-day averages in the Normative Aging Study [33], and with even longer averages in Multi-ethnic Study of Atherosclerosis [6]. However, several other observational studies have also found correlations between exposures and BP with shorter time lags [6,7,20]. In addition, in an cross-over randomized trial, Langrish et al. [34] showed that wearing a facemask for two hours to reduce air pollution exposure while walking in central Beijing reduced systolic BP. Differences in the study methods and design, levels of co-pollutants and their correlations with PM, and different characteristics of the study populations may account for the discrepancies in the results.

The inclusion of truck drivers and indoor office workers in our study was specifically designed to identify the effects of work-related traffic exposures on BP. However, in covariate-adjusted analyses we did not find any significant difference in BP between the two groups. Also, the levels of personal EC, a tracer of particle emissions from traffic, did not show any positive correlations with BP. In fact, EC showed a paradoxical negative association with diastolic and mean BP when the analysis was restricted to indoor office workers. Therefore, our results do not allow linking the effects of PM exposure on BP specifically to traffic emissions.

Our study had the advantage to have both personal and ambient measures of air pollution. All participants were evaluated with standard protocols for exposure assessment and measurement of BP. We conducted technical validation of personal PM_2.5 _measures that showed high reproducibility (r = 0.944) of our measurements. By measuring EC - a tracer of traffic particles - as well as by evaluating a group, i.e. truck drivers with direct exposure to traffic, we had the opportunity to distinguish the effects of traffic pollution from those of the general levels of ambient PM in Beijing. We also recognize that our study is subject to a number of limitations. Because of the relatively small sample size, we cannot exclude false negative findings as well as chance findings. Our results included some unexpected results, for instance, the finding of a negative association of EC with diastolic BP and mean arterial pressure among office workers. The literature regarding the association between BP and EC (or black carbon, [BC], which is highly correlated to EC) is limited and inconsistent. Mordukhovich et al. [33] found BC to be positively associated with systolic and diastolic BP in a cohort of elderly men. A study of 16 elderly subjects with respiratory disease showed no association between BC and blood pressure [19]. In a study with 62 cardiac rehabilitation patients, BC was positively associated with resting diastolic BP in single-pollutant models, but this association was found to be confounded by PM_2.5 _[9]. Further research is warranted to determine whether EC/BC is a determinant of increased BP. Our study was conducted in a short period of time in the summer of 2008. In a study of 10,459 individuals in South Korea, Choi et al. [7] showed stronger effects of PM exposure on BP during the warm season. Whether our findings can be extended to the winter season in Beijing remains to be determined. Although we used matching and multivariable models to control potential confounders, we cannot exclude residual confounding from measured and unmeasured variables, including different types of tea consumption and common activities conducted by the two groups during their work days. In addition to using personal PM_2.5 _and EC measures, we have utilized stationary measures of ambient PM_10 _to represent exposures. Simulation studies have shown that the error introduced by using data from stationary monitors is highly unlikely to bias away from the null, and indicated that this exposure misclassification may lead to an underestimation of the health effects of air pollution [35]. In addition, serial measures of ambient particulate concentrations have been shown to be representative of variations in personal exposures [36], particularly in the presence of high ambient PM levels [37].

## Conclusions

Our results showed a delayed effect of PM exposure on BP in individuals with high exposure to particulate pollution. The lack of associations with personal PM_2.5 _and EC measured during work hours indicates that effects on BP may be better captured with more protracted monitoring of air pollution levels in days before examination. Further investigations are warranted to estimate the impact of PM-related changes in BP on cardiovascular morbidity and mortality. Our results provide further support for the urgent implementation of measures for exposure reductions in the Beijing metropolitan area, as well as in areas with similarly high PM levels worldwide.

## List of abbreviations

BP: Blood Pressure; BTDAS: Beijing Truck Driver Air Pollution Study; CI: Confidence Interval; EC: Elemental Carbon; PM: Particulate Matter; PM_2.5_: particulate matter ≤2.5 μm; PM_10_: particulate matter ≤10 μm; SD: Standard Deviation.

## Competing interests

The authors declare that they have no competing interests.

## Authors' contributions

AB, SW, CD, PAB, JS, and LH designed the study and supervised the study operations. CD, JPM, AD, and XZ prepared the study protocols and oversaw their implementation. FB performed the statistical analyses. AB and LH wrote the manuscript. All authors provided edits and comments to the manuscript. All authors read and approved the final manuscript.

## Supplementary Material

Additional file 1 **Additional Tables Table S1 Stratified analyses by current smoking on all subjects (office workers and truck drivers) The results were stratified by current smoking for the associations of personal PM_2.5_, personal EC, and ambient PM_10 _with BP and heart rate for the entire study group**. Table S2 Stratified analyses by current smoking on office workers The results were stratified by current smoking for the associations of personal PM_2.5_, personal EC, and ambient PM_10 _with BP and heart rate for office workers. Table S3 Stratified analyses by current smoking on truck drivers The results were stratified by current smoking for the associations of personal PM_2.5_, personal EC, and ambient PM_10 _with BP and heart rate for truck drivers. Click here for file
