# Supplementary material for: Effects of particulate air pollution on blood pressure in a highly exposed population in Beijing, China: a repeated-measure study
Source: Environ Health. 2011 Dec 21;10:108. doi: 10.1186/1476-069X-10-108 (PMC3273442; doi:10.1186/1476-069X-10-108)
Supplement: Additional file 1 — Additional Tables Table S1 Stratified analyses by current smoking on all subjects (office workers and truck drivers) The results were stratified by current smoking for the associations of personal PM2.5, personal EC, and ambient PM10 with BP and heart rate for the entire study group. Table S2 Stratified analyses by current smoking on office workers The results were stratified by current smoking for the associations of personal PM2.5, personal EC, and ambient PM10 with BP and heart rate for office workers. Table S3 Stratified analyses by current smoking on truck drivers The results were stratified by current smoking for the associations of personal PM2.5, personal EC, and ambient PM10 with BP and heart rate for truck drivers. [file 1476-069X-10-108-S1.DOCX]

**Table S1. Stratified analyses by current smoking on all subjects (office workers and truck drivers)^a^**

|  |  |  | **All Subjects  (obs=240^b^)** | | |  | **Current non-smokers (obs=146^c^)** | | |  | **Current smokers  (obs=94^d^)** | | |
| --- | --- | --- | --- | --- | --- | --- | --- | --- | --- | --- | --- | --- | --- |
|  |  |  | β | (95%CI) | p-value |  | β | (95%CI) | p-value |  | β | (95%CI) | p-value |
|  |  |  |  |  |  |  |  |  |  |  |  |  |  |
| **Systolic blood pressure (mmHg)** | | | | | | | | | | | | | |
|  | Personal PM_2.5_ (work hours) |  | -0.01 | (-0.18;0.17) | 0.94 |  | 0.08 | (-0.26;0.41) | 0.64 |  | -0.04 | (-0.25;0.16) | 0.66 |
|  | Personal EC (work hours) |  | -0.29 | (-2.32;1.73) | 0.77 |  | -0.78 | (-3.72;2.16) | 0.60 |  | -0.95 | (-4.01;2.11) | 0.53 |
|  | Ambient PM_10_  (1-day mean) |  | 0.20 | (-0.05;0.45) | 0.11 |  | 0.10 | (-0.26;0.46) | 0.58 |  | 0.19 | (-0.12;0.49) | 0.22 |
|  | Ambient PM_10_  (2-day mean) |  | 0.26 | (-0.08;0.59) | 0.14 |  | 0.06 | (-0.44;0.56) | 0.81 |  | 0.06 | (-0.38;0.50) | 0.77 |
|  | Ambient PM_10_  (5-day mean) |  | 0.63 | (0.09;1.16) | 0.02 |  | 0.16 | (-0.61;0.92) | 0.69 |  | 0.51 | (0.17;1.18) | 0.14 |
|  | Ambient PM_10_  (8-day mean) |  | 0.98 | (0.34;1.61) | 0.003 |  | 0.33 | (-0.61;1.26) | 0.49 |  | 0.72 | (0.07;1.51) | 0.07 |
| **Diastolic blood pressure (mmHg)** | | | | | | | | | | | | | |
|  | Personal PM_2.5_ (work hours) |  | 0.04 | (-0.11;0.19) | 0.57 |  | 0.17 | (-0.10;0.45) | 0.22 |  | 0.04 | (-0.12;0.20) | 0.63 |
|  | Personal EC (work hours) |  | -1.26 | (-2.94;0.43) | 0.14 |  | -1.26 | (-3.82;1.29) | 0.33 |  | -1.19 | (-3.59;1.22) | 0.32 |
|  | Ambient PM_10_  (1-day mean) |  | 0.18 | (-0.03;0.39) | 0.09 |  | 0.25 | (-0.07;0.56) | 0.13 |  | 0.22 | (-0.17;0.61) | 0.26 |
|  | Ambient PM_10_  (2-day mean) |  | 0.17 | (-0.11;0.46) | 0.23 |  | 0.29 | (-0.15;0.73) | 0.19 |  | 0.24 | (-0.33;0.80) | 0.40 |
|  | Ambient PM_10_  (5-day mean) |  | 0.50 | (0.06;0.95) | 0.03 |  | 0.54 | (-0.10;1.18) | 0.09 |  | 0.89 | (0.04;1.74) | 0.04 |
|  | Ambient PM_10_  (8-day mean) |  | 0.71 | (0.18;1.24) | 0.01 |  | 0.85 | (0.08;1.63) | 0.03 |  | 1.55 | (0.59;2.51) | 0.002 |
| **Mean arterial pressure (mmHg)** | | | | | | | | | | | | | |
|  | Personal PM_2.5_ (work hours) |  | 0.03 | (-0.11;0.17) | 0.66 |  | 0.14 | (-0.12;0.40) | 0.29 |  | 0.02 | (-0.14;0.17) | 0.81 |
|  | Personal EC (work hours) |  | -0.94 | (-2.52;0.63) | 0.24 |  | -1.35 | (-3.68;0.99) | 0.25 |  | -0.92 | (-3.23;1.40) | 0.43 |
|  | Ambient PM_10_  (1-day mean) |  | 0.20 | (0.01;0.39) | 0.04 |  | 0.22 | (-0.08;0.51) | 0.14 |  | 0.20 | (-0.09;0.50) | 0.17 |
|  | Ambient PM_10_  (2-day mean) |  | 0.20 | (-0.07;0.46) | 0.14 |  | 0.21 | (-0.19;0.62) | 0.29 |  | 0.13 | (-0.30;0.55) | 0.56 |
|  | Ambient PM_10_  (5-day mean) |  | 0.55 | (0.13;0.96) | 0.01 |  | 0.40 | (-0.20;0.99) | 0.19 |  | 0.65 | (0.00;1.30) | 0.05 |
|  | Ambient PM_10_  (8-day mean) |  | 0.81 | (0.31;1.30) | 0.002 |  | 0.69 | (-0.04;1.42) | 0.06 |  | 1.02 | (0.27;1.77) | 0.01 |
| **Pulse pressure (mmHg)** | | | | | | | | | | | | | |
|  | Personal PM_2.5_ (work hours) |  | -0.06 | (-0.22;0.10) | 0.49 |  | -0.04 | (-0.34;0.27) | 0.80 |  | -0.09 | (-0.28;0.09) | 0.30 |
|  | Personal EC (work hours) |  | 0.75 | (-1.1;2.6) | 0.42 |  | 0.49 | (-2.38;3.35) | 0.73 |  | -0.06 | (-2.71;2.59) | 0.97 |
|  | Ambient PM_10_  (1-day mean) |  | 0.01 | (-0.22;0.23) | 0.96 |  | -0.14 | (-0.49;0.20) | 0.41 |  | 0.05 | (-0.29;0.38) | 0.77 |
|  | Ambient PM_10_  (2-day mean) |  | 0.10 | (-0.21;0.41) | 0.51 |  | -0.13 | (-0.61;0.36) | 0.60 |  | 0.19 | (-0.28;0.66) | 0.43 |
|  | Ambient PM_10_  (5-day mean) |  | 0.12 | (-0.38;0.61) | 0.64 |  | -0.24 | (-0.96;0.48) | 0.50 |  | 0.36 | (-0.38;1.10) | 0.33 |
|  | Ambient PM_10_  (8-day mean) |  | 0.22 | (-0.37;0.81) | 0.46 |  | -0.47 | (-1.33;0.39) | 0.28 |  | 0.79 | (-0.06;1.64) | 0.07 |
| **Heart rate (bpm)** | | | | | | | | | | | | | |
|  | Personal PM_2.5_ (work hours) |  | 0.15 | (-0.08;0.39) | 0.20 |  | 0.35 | (-0.06;0.75) | 0.09 |  | 0.00 | (-0.30;031) | 0.97 |
|  | Personal EC (work hours) |  | 1.03 | (-1.62;3.68) | 0.44 |  | 2.28 | (-1.60;6.17) | 0.24 |  | -2.91 | (-6.85;1.04) | 0.14 |
|  | Ambient PM_10_  (1-day mean) |  | 0.14 | (-0.19;0.48) | 0.40 |  | 0.42 | (-0.06;0.91) | 0.09 |  | -0.21 | (-0.75;0.34) | 0.44 |
|  | Ambient PM_10_  (2-day mean) |  | 0.23 | (-0.23;0.68) | 0.33 |  | 0.44 | (-0.22;1.11) | 0.19 |  | -0.36 | (-1.12;0.40) | 0.34 |
|  | Ambient PM_10_  (5-day mean) |  | 0.57 | (-0.14;1.29) | 0.12 |  | 0.82 | (-0.14;1.79) | 0.09 |  | -0.11 | (-1.31;1.10) | 0.86 |
|  | Ambient PM_10_  (8-day mean) |  | 0.67 | (-0.20;1.53) | 0.13 |  | 0.88 | (-0.33;2.10) | 0.15 |  | 0.03 | (-1.36;1.42) | 0.97 |

^a^All analyses adjusted for age, sex, BMI, pack-years tea drinking during the study time, usual alcohol drinking, work hours/week, day of the week, and appropriate outdoor temperature (i.e., temperature averaged over the same time window as the air particle exposure variable). Analyses on all subjects are also adjusted for smoking status (current, ex, never) and number of cigarettes smoked during the study time. Analyses on current smokers are also adjusted for number of cigarettes smoked during the study time. ^b^For EC, results are estimated on 238 observations because of two missing values; for PM_2.5_, results are from 239 observations because of one missing value. ^c^For PM_2.5_ and EC exposures, results are estimated on 145 observations because of a missing value. ^d^For EC exposures, results are estimated on 93 observations because of a missing value.

**Table S2. Stratified analyses by current smoking on office workers^a^**

|  |  |  | **All Office Workers (obs=120^b^)** | | |  | **Current non-smoking office workers (obs=74^c^)** | | |  | **Current smoking office workers (obs=46^d^)** | | |
| --- | --- | --- | --- | --- | --- | --- | --- | --- | --- | --- | --- | --- | --- |
|  |  |  | β | (95%CI) | p-value |  | β | (95%CI) | p-value |  | β | (95%CI) | p-value |
|  |  |  |  |  |  |  |  |  |  |  |  |  |  |
| **Systolic blood pressure (mmHg)** | | | | | | | | | | | | | |
|  | Personal PM_2.5_ (work hours) |  | -0.06 | (-0.29;0.18) | 0.64 |  | -0.13 | (-0.60;0.35) | 0.59 |  | -0.03 | (-0.32;0.27) | 0.86 |
|  | Personal EC (work hours) |  | -2.54 | (-6.39;1.31) | 0.19 |  | -4.74 | (-10.37;0.89) | 0.10 |  | -5.72 | (-13.15;1.71) | 0.12 |
|  | Ambient PM_10_  (1-day mean) |  | 0.10 | (-0.26;0.46) | 0.57 |  | -0.25 | (-0.77;0.27) | 0.33 |  | 0.18 | (-0.32;0.67) | 0.46 |
|  | Ambient PM_10_  (2-day mean) |  | -0.05 | (-0.53;0.44) | 0.85 |  | -0.40 | (-1.09;0.29) | 0.25 |  | -0.12 | (-0.84;0.61) | 0.73 |
|  | Ambient PM_10_  (5-day mean) |  | 0.08 | (-0.80;0.95) | 0.86 |  | -0.26 | (-1.38;0.85) | 0.63 |  | -0.17 | (-1.59;1.25) | 0.80 |
|  | Ambient PM_10_  (8-day mean) |  | 0.53 | (-0.44;1.50) | 0.28 |  | -0.18 | (-1.44;1.08) | 0.77 |  | 0.36 | (-1.41;2.13) | 0.67 |
| **Diastolic blood pressure (mmHg)** | | | | | | | | | | | | | |
|  | Personal PM_2.5_ (work hours) |  | 0.00 | (-0.21;0.22) | 0.97 |  | -0.06 | (-0.52;0.40) | 0.79 |  | 0.04 | (-0.19;0.26) | 0.73 |
|  | Personal EC (work hours) |  | -4.52 | (-7.87;-1.16) | 0.01 |  | -4.69 | (-10.28;0.89) | 0.10 |  | -5.47 | (-10.89;-0.04) | 0.05 |
|  | Ambient PM_10_  (1-day mean) |  | 0.11 | (-0.21;0.43) | 0.49 |  | -0.04 | (-0.58;0.50) | 0.89 |  | 0.32 | (-0.37;1.02) | 0.34 |
|  | Ambient PM_10_  (2-day mean) |  | 0.06 | (-0.38;0.50) | 0.78 |  | 0.11 | (-0.60;0.82) | 0.75 |  | -0.01 | (-1.02;1.00) | 0.98 |
|  | Ambient PM_10_  (5-day mean) |  | 0.31 | (-0.43;1.06) | 0.40 |  | 0.74 | (-0.31;1.78) | 0.16 |  | 0.61 | (-1.41;2.62) | 0.53 |
|  | Ambient PM_10_  (8-day mean) |  | 0.83 | (0.02;1.64) | 0.04 |  | 1.39 | (0.22;2.56) | 0.02 |  | 1.60 | (-0.86;4.06) | 0.18 |
| **Mean arterial pressure (mmHg)** | | | | | | | | | | | | | |
|  | Personal PM_2.5_ (work hours) |  | -0.01 | (-0.19;0.18) | 0.95 |  | -0.07 | (-0.46;0.33) | 0.74 |  | 0.02 | (-0.19;0.23) | 0.84 |
|  | Personal EC (work hours) |  | -3.74 | (-6.70;-0.78) | 0.01 |  | -4.70 | (-9.37;-0.04) | 0.05 |  | -5.06 | (-10.22;0.10) | 0.05 |
|  | Ambient PM_10_  (1-day mean) |  | 0.12 | (-0.16;0.41) | 0.39 |  | -0.08 | (-0.54;0.38) | 0.73 |  | 0.23 | (-0.25;0.72) | 0.32 |
|  | Ambient PM_10_  (2-day mean) |  | 0.05 | (-0.34;0.44) | 0.81 |  | 0.00 | (-0.60;0.60) | 1.00 |  | -0.07 | (-0.79;0.65) | 0.84 |
|  | Ambient PM_10_  (5-day mean) |  | 0.27 | (-0.42;0.95) | 0.44 |  | 0.47 | (0.46;1.40) | 0.31 |  | 0.08 | (-1.34;1.51) | 0.90 |
|  | Ambient PM_10_  (8-day mean) |  | 0.74 | (0.00;1.48) | 0.05 |  | 0.90 | (-0.16;1.96) | 0.09 |  | 0.78 | (-0.97;2.54) | 0.35 |
| **Pulse pressure (mmHg)** | | | | | | | | | | | | | |
|  | Personal PM_2.5_ (work hours) |  | -0.06 | (-0.31;0.20) | 0.65 |  | -0.03 | (-0.52;0.47) | 0.91 |  | -0.07 | (-0.37;0.22) | 0.59 |
|  | Personal EC (work hours) |  | 2.69 | (-1.42;6.8) | 0.19 |  | 0.19 | (-6.14;6.53) | 0.95 |  | 0.15 | (-7.38;7.68) | 0.97 |
|  | Ambient PM_10_  (1-day mean) |  | -0.03 | (-0.40;0.33) | 0.86 |  | -0.18 | (-0.77;0.40) | 0.52 |  | 0.14 | (-0.49;0.76) | 0.65 |
|  | Ambient PM_10_  (2-day mean) |  | -0.10 | (-0.60;0.40) | 0.68 |  | -0.39 | (-1.18;0.40) | 0.32 |  | 0.10 | (-0.79;0.99) | 0.81 |
|  | Ambient PM_10_  (5-day mean) |  | -0.26 | (-1.13;0.60) | 0.54 |  | -0.93 | (-2.07;0.21) | 0.10 |  | 0.79 | (-0.96;2.54) | 0.35 |
|  | Ambient PM_10_  (8-day mean) |  | -0.38 | (-1.35;0.59) | 0.44 |  | -1.61 | (-2.84;-0.38) | 0.01 |  | 1.31 | (-0.86;3.48) | 0.22 |
| **Heart rate (bpm)** | | | | | | | | | | | | | |
|  | Personal PM_2.5_ (work hours) |  | 0.00 | (-0.31;0.30) | 0.97 |  | -0.03 | (-0.64;0.50) | 0.92 |  | 0.09 | (-0.30;0.47) | 0.63 |
|  | Personal EC (work hours) |  | -2.08 | (-6.94;2.79) | 0.40 |  | 1.28 | (-8.47;5.91) | 0.72 |  | 2.17 | (-6.56;10.91) | 0.60 |
|  | Ambient PM_10_  (1-day mean) |  | -0.03 | (-0.48;0.41) | 0.88 |  | 0.12 | (-0.56;0.80) | 0.71 |  | -0.39 | (-1.15;0.36) | 0.28 |
|  | Ambient PM_10_  (2-day mean) |  | -0.14 | (-0.73;0.46) | 0.64 |  | -0.26 | (-1.15;0.63) | 0.55 |  | -0.16 | (-1.25;0.92) | 0.75 |
|  | Ambient PM_10_  (5-day mean) |  | -0.13 | (-1.14;0.89) | 0.81 |  | -0.05 | (-1.39;1.30) | 0.94 |  | 0.13 | (-1.81;2.07) | 0.89 |
|  | Ambient PM_10_  (8-day mean) |  | -0.44 | (-1.59;0.71) | 0.45 |  | -0.30 | (-1.90;1.30) | 0.70 |  | -0.92 | (-3.31;1.47) | 0.42 |

^a^Adjusted for age, sex, BMI, pack-years, tea drinking during the study time, usual alcohol drinking, work hours/week, day of the week, and appropriate outdoor temperature (i.e., temperature averaged over the same time window as the air particle exposure variable). Analyses on all subjects are also adjusted for smoking status (current, ex, never) and number of cigarettes smoked during the study time. Analyses on current smokers are also adjusted for number of cigarettes smoked during the study time. ^b^For EC exposure, results are estimated on 119 observations because of two missing values. ^c^For EC, results are estimated on 73 observations because of a missing values. ^d^For EC, results are estimated on 45 observations because of a missing value.

**Table S3. Stratified analyses by current smoking on truck drivers^a^**

|  |  |  | **All truck drivers (obs=120^b^)** | | |  | **Current non-smoking truck drivers (obs=72^c^)** | | |  | **Current smoking truck drivers (obs=48)** | | |
| --- | --- | --- | --- | --- | --- | --- | --- | --- | --- | --- | --- | --- | --- |
|  |  |  | β | (95%CI) | p-value |  | β | (95%CI) | p-value |  | β | (95%CI) | p-value |
|  |  |  |  |  |  |  |  |  |  |  |  |  |  |
| **Systolic blood pressure (mmHg)** | | | | | | | | | | | | | |
|  | Personal PM_2.5_ (work hours) |  | 0.15 | (-0.16;0.46) | 0.33 |  | 0.29 | (-0.34;0.93) | 0.35 |  | 0.16 | (-0.31;0.62) | 0.48 |
|  | Personal EC (work hours) |  | 1.23 | (-1.53;3.99) | 0.38 |  | 0.77 | (-3.75;5.28) | 0.73 |  | 1.23 | (-3.18;5.65) | 0.56 |
|  | Ambient PM_10_  (1-day mean) |  | 0.24 | (-0.13;0.60) | 0.20 |  | 0.14 | (-0.40;0.68) | 0.60 |  | 0.22 | (-0.19;0.62) | 0.27 |
|  | Ambient PM_10_  (2-day mean) |  | 0.47 | (-0.04;0.97) | 0.07 |  | 0.23 | (-0.55;1.01) | 0.55 |  | 0.19 | (-0.44;0.83) | 0.53 |
|  | Ambient PM_10_  (5-day mean) |  | 0.97 | (0.15;1.78) | 0.02 |  | 0.20 | (-1.07;1.46) | 0.75 |  | 0.99 | (0.13;1.84) | 0.03 |
|  | Ambient PM_10_  (8-day mean) |  | 1.31 | (0.32;2.31) | 0.01 |  | 0.61 | (-0.98;2.20) | 0.44 |  | 0.51 | (-0.49;1.50) | 0.29 |
| **Diastolic blood pressure (mmHg)** | | | | | | | | | | | | | |
|  | Personal PM_2.5_ (work hours) |  | 0.09 | (-0.14;0.33) | 0.42 |  | 0.30 | (-0.16;0.76) | 0.19 |  | 0.16 | (-0.16;0.48) | 0.31 |
|  | Personal EC (work hours) |  | 0.23 | (-1.84;2.3) | 0.83 |  | 0.34 | (-2.98;-3.66) | 0.83 |  | 0.29 | (-2.94;3.52) | 0.85 |
|  | Ambient PM_10_  (1-day mean) |  | 0.24 | (-0.03;0.51) | 0.08 |  | 0.31 | (-0.08;0.70) | 0.12 |  | 0.03 | (-0.56;0.62) | 0.92 |
|  | Ambient PM_10_  (2-day mean) |  | 0.15 | (-0.22;0.53) | 0.42 |  | 0.11 | (-0.45;0.67) | 0.69 |  | 0.21 | (-0.70;1.11) | 0.63 |
|  | Ambient PM_10_  (5-day mean) |  | 0.34 | (-0.29;0.97) | 0.28 |  | -0.58 | (-1.47;0.31) | 0.19 |  | 1.08 | (-0.21;2.37) | 0.09 |
|  | Ambient PM_10_  (8-day mean) |  | 0.07 | (-0.72;0.87) | 0.86 |  | -0.74 | (-1.89;0.40) | 0.19 |  | 1.24 | (-0.23;2.71) | 0.09 |
| **Mean arterial pressure (mmHg)** | | | | | | | | | | | | | |
|  | Personal PM_2.5_ (work hours) |  | 0.12 | (-0.11;0.35) | 0.30 |  | 0.29 | (-0.17;0.75) | 0.21 |  | 0.16 | (-0.17;0.48) | 0.32 |
|  | Personal EC (work hours) |  | 0.51 | (-1.55;2.57) | 0.62 |  | 0.31 | (-3.01;-3.62) | 0.85 |  | 0.58 | (-2.64;3.80) | 0.70 |
|  | Ambient PM_10_  (1-day mean) |  | 0.25 | (-0.02;0.51) | 0.07 |  | 0.26 | (-0.12;0.65) | 0.17 |  | 0.16 | (-0.26;0.57) | 0.44 |
|  | Ambient PM_10_  (2-day mean) |  | 0.25 | (-0.13;0.62) | 0.19 |  | 0.12 | (-0.43;0.67) | 0.65 |  | 0.20 | (-0.45;0.84) | 0.53 |
|  | Ambient PM_10_  (5-day mean) |  | 0.56 | (-0.05;1.17) | 0.07 |  | -0.35 | (-1.24;0.53) | 0.42 |  | 1.06 | (0.22;1.91) | 0.02 |
|  | Ambient PM_10_  (8-day mean) |  | 0.48 | (-0.29;1.26) | 0.22 |  | -0.31 | (-1.46;0.85) | 0.59 |  | 0.79 | (-0.21;1.80) | 0.11 |
| **Pulse pressure (mmHg)** | | | | | | | | | | | | | |
|  | Personal PM_2.5_ (work hours) |  | 0.06 | (-0.20;0.31) | 0.65 |  | 0.17 | (-0.34;0.69) | 0.50 |  | 0.23 | (-0.17;0.63) | 0.24 |
|  | Personal EC (work hours) |  | 0.98 | (-1.18;3.14) | 0.37 |  | 1.38 | (-2.22;4.97) | 0.44 |  | 2.17 | (-1.14;5.48) | 0.18 |
|  | Ambient PM_10_  (1-day mean) |  | 0.02 | (-0.28;0.32) | 0.90 |  | -0.16 | (-0.61;0.29) | 0.47 |  | -0.11 | (-0.61;0.40) | 0.66 |
|  | Ambient PM_10_  (2-day mean) |  | 0.33 | (-0.08;0.75) | 0.11 |  | 0.15 | (-0.52;0.83) | 0.64 |  | 0.26 | (-0.49;1.01) | 0.47 |
|  | Ambient PM_10_  (5-day mean) |  | 0.56 | (-0.13;1.26) | 0.11 |  | 0.71 | (-0.34;1.76) | 0.18 |  | 0.16 | (-1.06;1.39) | 0.78 |
|  | Ambient PM_10_  (8-day mean) |  | 1.08 | (0.27;1.89) | 0.01 |  | 1.18 | (-0.08;2.45) | 0.07 |  | 0.67 | (-0.62;1.95) | 0.28 |
| **Heart rate (bpm)** | | | | | | | | | | | | | |
|  | Personal PM_2.5_ (work hours) |  | 0.30 | (-0.14;0.74) | 0.18 |  | 0.37 | (-0.45;1.19) | 0.36 |  | 0.16 | (-0.51;0.83) | 0.61 |
|  | Personal EC (work hours) |  | 2.19 | (-1.52;5.89) | 0.24 |  | 1.75 | (-3.82;7.32) | 0.52 |  | -0.11 | (-6.11;5.89) | 0.97 |
|  | Ambient PM_10_  (1-day mean) |  | 0.29 | (-0.23;0.81) | 0.27 |  | 0.38 | (-0.35;1.12) | 0.29 |  | -0.15 | (-1.05;0.75) | 0.73 |
|  | Ambient PM_10_  (2-day mean) |  | 0.61 | (-0.13;1.35) | 0.10 |  | 0.40 | (-0.73;1.53) | 0.47 |  | -0.30 | (-1.62;1.01) | 0.63 |
|  | Ambient PM_10_  (5-day mean) |  | 1.20 | (-0.02;2.42) | 0.05 |  | 1.09 | (-0.68;2.86) | 0.22 |  | 0.28 | (-1.82;2.38) | 0.78 |
|  | Ambient PM_10_  (8-day mean) |  | 1.69 | (0.27;3.12) | 0.02 |  | 1.40 | (-0.78;3.59) | 0.20 |  | 0.77 | (-1.47;3.01) | 0.47 |

^a^Adjusted for age, sex, BMI, pack-years, number of cigarettes smoked and tea drinking during the study time, usual alcohol drinking, work hours/week, day of the week, and appropriate outdoor temperature (i.e., temperature averaged over the same time window as the air particle exposure variable). Analyses on all subjects are also adjusted for smoking status (current, ex, never) and number of cigarettes smoked during the study time. Analyses on current smokers are also adjusted for number of cigarettes smoked during the study time. ^b^For PM_2.5_ and EC exposures, results are estimated on 119 observations because of a missing value. ^c^For PM_2.5_, results are from 71 observations because of a missing value.
